# Effects of NH_3_ Plasma and Mg Doping on InGaZnO pH Sensing Membrane

**DOI:** 10.3390/membranes11120994

**Published:** 2021-12-20

**Authors:** Chyuan-Haur Kao, Chia-Shao Liu, Shih-Ming Chan, Chih-Chen Kuo, Shang-Che Tsai, Ming-Ling Lee, Hsiang Chen

**Affiliations:** 1 Department of Electronic Engineering, Chang Gung University, 259 Wen-Hwa 1st Road, Kwei-Shan, Taoyuan 333, Taiwan; chkao@mail.cgu.edu.tw (C.-H.K.); chkao@mail.cgu.edu (C.-S.L.); 2 Kidney Research Center, Department of Nephrology, Chang Gung Memorial Hospital, Chang Gung University, No.5, Fuxing St., Guishan Township, Taoyuan 333, Taiwan; 3 Department of Electronic Engineering, Ming Chi University of Technology, 284 Gungjuan Rd., Taishan Dist., New Taipei City 24301, Taiwan; 4 Department of Applied Materials and Optoelectronic Engineering, National Chi Nan University, Puli, Nantou 545, Taiwan; s107328009@mail1.ncnu.edu.tw (S.-M.C.); s107328003@mail1.ncnu.edu.tw (C.-C.K.); s1063280042@mail1.ncnu.edu.tw (S.-C.T.); 5 Department of Electro-Optical Engineering, Minghsin University of Science and Technology, No.1, Xinxing Road, Xinfeng, Hsinchu 304, Taiwan

**Keywords:** indium gallium zinc oxide, magnesium doped, membranes, sputtering, ammonia plasma treatment, pH sensing, biosensors

## Abstract

In this study, the effects of magnesium (Mg) doping and Ammonia (NH_3_) plasma on the pH sensing capabilities of InGaZnO membranes were investigated. Undoped InGaZnO and Mg-doped pH sensing membranes with NH_3_ plasma were examined with multiple material analyses including X-ray diffraction, X-ray photoelectron spectroscopy, secondary ion mass spectroscopy and transmission electron microscope, and pH sensing behaviors of the membrane in electrolyte-insulator-semiconductors. Results indicate that Mg doping and NH_3_ plasma treatment could superpositionally enhance crystallization in fine nanostructures, and strengthen chemical bindings. Results indicate these material improvements increased pH sensing capability significantly. Plasma-treated Mg-doped InGaZnO pH sensing membranes show promise for future pH sensing biosensors.

## 1. Introduction

The ion sensitive field-effect transistor (ISFET) with a sensing membrane for use in bio-sensing applications was demonstrated by P. Bergveld in 1970 [1]. When integrated with complementary metal oxide semiconductor technology, ISFETs are capable of detecting ion activity in the human body [2]. Concentrations of biologically related ions such H^+^, Na^+^, and K^+^ are important health indexes associated with disease monitoring. Recently, various high-k dielectrics such as Ta_2_O_5_ [3], Pr_2_O_3_ [4], and Er_2_O_3_ [5] have been proposed as sensing membrane materials to detect these crucial ions in the human body [6,7]. To further enhance ion-sensing capabilities, it is worthwhile to explore materials, alternative fabrication processes, and treatments such as addition of nanoparticles or modulation of the membrane thickness [8,9]. In addition, incorporating post treatments such as Magnesium (Mg) doping and ammonia (NH_3_) plasma treatment may optimize the membrane performance [7,10,11]. In this study, InGaZnO films, which can function as a transparent conductive oxide, have been demonstrated as ion-sensing membranes in electrolyte-insulator-semiconductor (EIS) structures. Furthermore, magnesium atoms were doped into InGaZnO films and NH_3_ plasma treatment was incorporated into the membrane fabrication process [12,13] to boost ion-sensing behavior. Based on previous studies [10,14,15], magnesium atoms can fill in vacancies and reduce dangling bonds, and NH_3_ plasma treatment can include N atoms into the membrane to mitigate defects. Therefore, combination of Mg doping and NH_3_ plasma treatment may effectively improve material quality and ion-sensing capability [13]. To examine an InGaZnO membrane with these treatments, multiple material characterization techniques were performed, and sensing behaviors were evaluated [16,17]. To observe the morphologies both on the surface and in the cross section, scanning electron microscopy (SEM), atomic force microscopy (AFM), and transmission electron microscopy (TEM) images were taken. X-ray diffraction (XRD) was used to monitor crystalline structures, X-ray photoelectron spectroscopy (XPS) was used to study chemical bindings, and secondary ion mass spectrometry (SIMS) was used to evaluate element composition along the depth. Results indicate that Mg doping and NH_3_ plasma treatment could include both Mg and N atoms into the membrane to reinforce the chemical binding and strengthen crystallization [18]. Notably, AFM and SEM images revealed grainization on the membrane surface, and TEM images indicated passivation of cracks and separation lines in the cross section.

Consistent with material improvements, pH sensing capability was enhanced, and hysteresis voltage and drift rate were suppressed with the incorporation of Mg doping and NH3 plasma treatment in the membrane fabrication process [19,20]. This study confirms that incorporation of Mg doping and NH3 plasma treatment can work together to optimize membrane material properties and sensing performance [21,22]. Furthermore, based our previous study [23], appropriate annealing could effective improve the material quality and sensing behaviors. In this study, different from the annealing treatment, compared with the as-deposited samples, NH3 plasma treatment could boost InGaZnO samples with or without Mg doping in terms of sensing capability more effectively. Therefore, NH3 plasma treatment in this study is more favorable than annealing treatment in terms of improving the pH sensing behaviors. InGaZnO membranes with Mg doping and NH3 plasma treatment can achieve excellent sensing performance and are promising for fabrication of future portable biosensing devices [24].

## 2. Experimental

**Figure 1:**
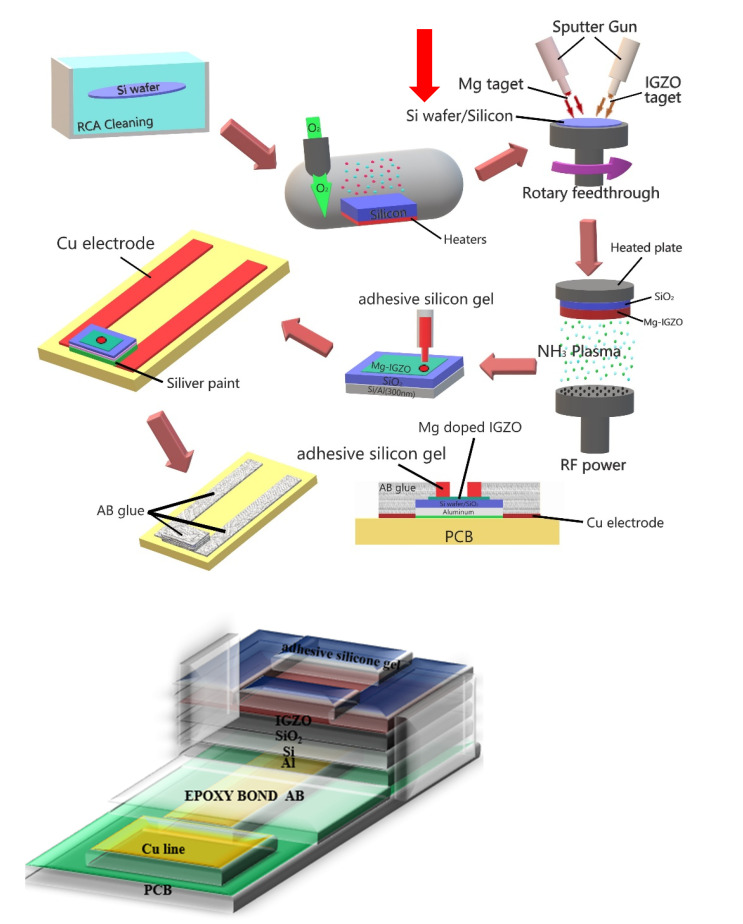
Schematic diagram of fabrication processes of Mg doped IGZO membranes with NH_3_ plasma treatment in EIS structures.

To fabricate InGaZnO sensing membranes on electrolyte-insulator-semiconductor structures, the structure was deposited on 4-inch n-type (100) silicon wafers with resistivity of 5–10 Ω-cm [25]. To remove any native oxide, the wafers were cleaned using HF (HF:H_2_O = 1:100). Next, 50-nm SiO_2_ was grown by thermal wet oxidation. In our experiment, InGaZnO and Mg targets were purchased from Gredmann Company, Taiwan. Then, in the condition, 50-nm InGaZnO was deposited on the wafer by radio frequency (RF) reactive sputtering with a mixture of Ar and O_2_ (Ar:O_2_ = 23:2) ambient during sputtering [23]. In the second condition, a 50-nm Mg-doped InGaZnO sensing membrane was deposited by co-sputtering on the wafer. During the reactive sputtering, InGaZnO and Mg targets were used in an ambient of Ar:O_2_ at 23:2 with RF power at 80 W and ambient pressure of 1.3 Pa. Both undoped and Mg-doped samples were subjected to a post-NH_3_ plasma treatment in a plasma-enhanced chemical vapor deposition (PECVD) system with an RF power of 30 W for 1 min and 3 min, respectively. The NH_3_ plasma treatment was performed by a PECVD and the model and make of the PECVD was Sancom and PD-240. An Al film 300 nm in thickness was then deposited on the backside of the wafer. Next, adhesive silicone gel was used to define a sensing window. Finally, the samples were fabricated in silver gel on the copper lines of a printed circuit board. An epoxy package was used to separate the EIS structure and the copper lines. Since the EIS structure is not stable because defects between interface layer and silicon. To overcome the problem, incorporation of NH_3_ plasma treatement were used to optimize the sensing performance. The detailed fabrication process is illustrated in Figure 1.

To evaluate the sensing behavior of a membrane, the ionic consumption reactions between the solution/sensing membrane can be explained by the site-binding model [26,27,28]. The voltage of the surface potential (*ψ*) depends on the pH concentration of the electrolyte and the sensing factor *β*. The value of (*ψ*) can be figured out using Equation (1).

(1) $$\psi =2.303\frac{kT}{q}\frac{\beta }{\beta +1}(pHpzc-pH)$$

(*k* is Boltzmann’s constant, *T* is the temperature, *q* is the elementary charge, *pH_pzc_* is the *pH* value with no charge). *β* is a factor related to the sensitivity of the gate membrane. Furthermore, the *β* is linked to the density of surface hydroxyl groups, as described in Equation (2). *N_s_* is the number of surface site/area and *C_DL_* is the double layer capacitance based on the Gouy–Chapman–Stern model [29].

(2) $$\beta =\frac{2q^{2}N_{s}\sqrt{K_{a}K_{b}}}{kTC_{DL}}$$

To analyze the membrane films, multiple material analyzing techniques are performed. The make and model of SEM and TEM are JEOL JSM-7500F and JEOL JEM 2100 PLUS, respectively. The operating voltages for SEM and TEM were 15 kV and 200 kV, respectively. In addition, the make and model of PL is HITACHI F-4500. The excitation laser wavelength was 325 nm with a laser spot diameter of 1 μm. The PL spectral range was 330~1000 nm (CCD sensor) and 1000~1500 nm (InGaAs sensor). As for the XRD apparatus, the make and model is Bruker D8 Discover. For XRD analysis of the samples, the grazing incidence of X-ray beam CuKa (*k* = 1.542  Å) radiation is used with an incidence angle step of 0.5° in the diffraction angle range

(2θ) from 20° to 60°. The make and model of SIMS was CAMECA IMS-7f with O^2+^ ion source and image resolution ≒ 10,000, mass range ≒ 300. The SIMS instrument was used by an internally yielded beam ions focused on a sample surface to produce secondary ions. The generated ions were then passed through a mass spectrometer across a high electrostatic potential. The depth profiles of elemental and molecular species could be assessed by SIMS analysis. The AFM model and make are Bruker Dimension Icon. The image resolution of X-Y noise was less than 0.15 nm and Z noise less than 30 pm (Close Loop). The scanning range (X,Y) was 1 μm ∗ 1 μm. The AFM in Bruker Dimension Icon modes with intermittent contact was using a silicon tip with a 10 pN/nm spring constant. A sample area of 3 × 3 μm was scanned with actuation rates up to 8 kHz in air and fluid.

The XPS model and make are XPS ULVAC-PHI and PHI 5000. The XPS spectra were carried out by a VG ESCA Scientific Theta Probe. As for the XPS instrument condition, the X-ray spot size was about 15 μm, the take-off angle was around 53° and the pass energy was set as 50 eV. The X-ray source for the XPS measurement was Al Kα (1486.6 eV). Furthermore, the sputtering argon ion beam with a beam energy of 3 kV was operated at a current density of 1 μA/mm^2^.

## 3. Results and Discussion

**Figure 2:**
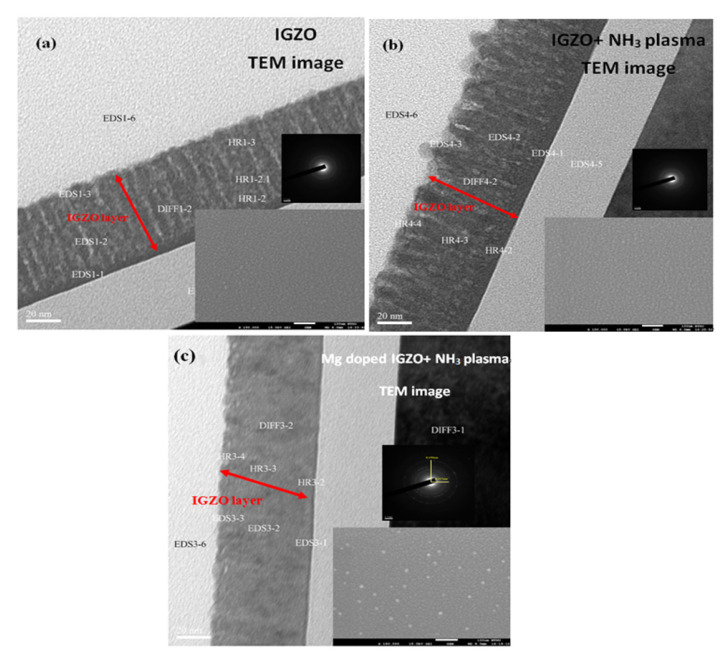
TEM images with SAED, FESEM sub-images for (**a**) IGZO sample (**b**) IGZO with NH_3_ plasma treatment (**c**) Mg doped IGZO with NH_3_ plasma treatment.

**Figure 3:**
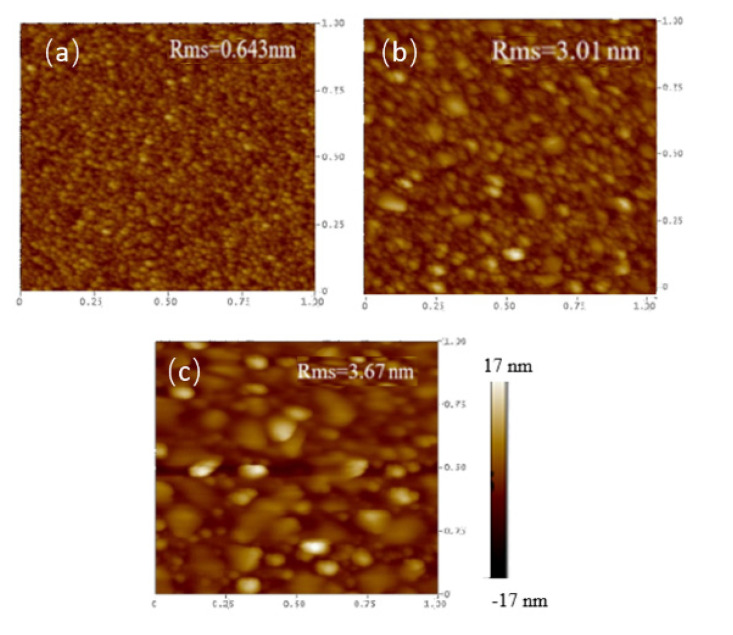
AFM images for (**a**) IGZO sample (**b**) IGZO with NH_3_ plasma treatment (**c**) Mg doped IGZO with NH_3_ plasma treatment.

In this study, the as-deposited InGaZnO samples were subjected to the NH_3_ plasma treatment, material characterizations and sensing measurements were performed on the as-deposited samples and the samples with NH_3_ plasma treatment. To examine the surface morphologies both on the surface and through the cross section, SEM images on the surface and TEM images on the cross section of the InGaZnO sample were taken. The InGaZnO sample with NH_3_ plasma treatment and the Mg-doped InGaZnO sample with plasma treatment are shown in Figure 2 and Figure 3. Both AFM and SEM images revealed that some crystals were generated on the surface of the samples treated with NH_3_ plasma compared with the untreated InGaZnO samples. Moreover, line-shaped stripes in the untreated InGaZnO sample were reduced by NH_3_ plasma treatment, and even eliminated by Mg doping plus NH_3_ plasma treatment [3], as shown in Figure 2 and Figure 3. Based on previous studies, NH_3_ plasma treatment can roughen the surface and hence enhance the crystallization and grain size [11]. Therefore, the sensing factor β could be increased with the increased roughness value as shown on the AFM images (Figure 3) because the number of sites exposed to the solution was enlarged. Moreover, the N atoms caused by NH_3_ plasma incorporated into the film could mitigate the dangling bonds and reduce the traps [13].

**Figure 4:**
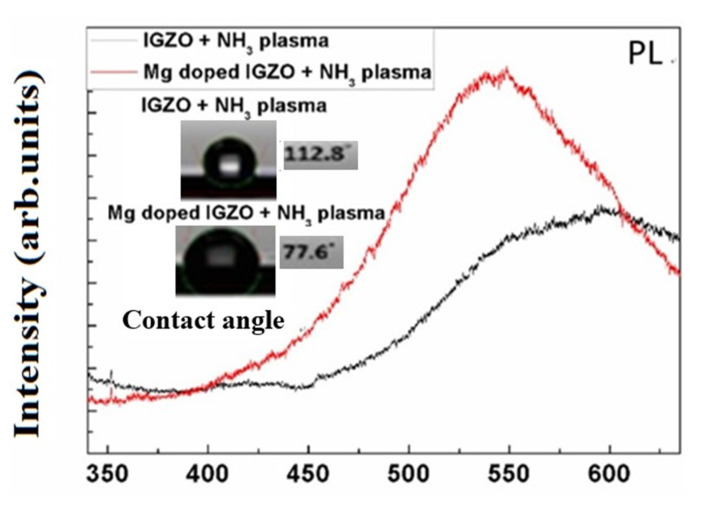
PL spectra and surface contact angle measurements for IGZO with NH_3_ plasma treatment and Mg doped IGZO with NH_3_ plasma treatment.

Furthermore, the photoluminescence (PL) peak was enhanced and blue-shifted, which may result from crystallization. In addition, contact angle measurements revealed that the enhancement of hydrophilic properties might result from modification of the surface due to Mg doping, as shown in Figure 4.

**Figure 5:**
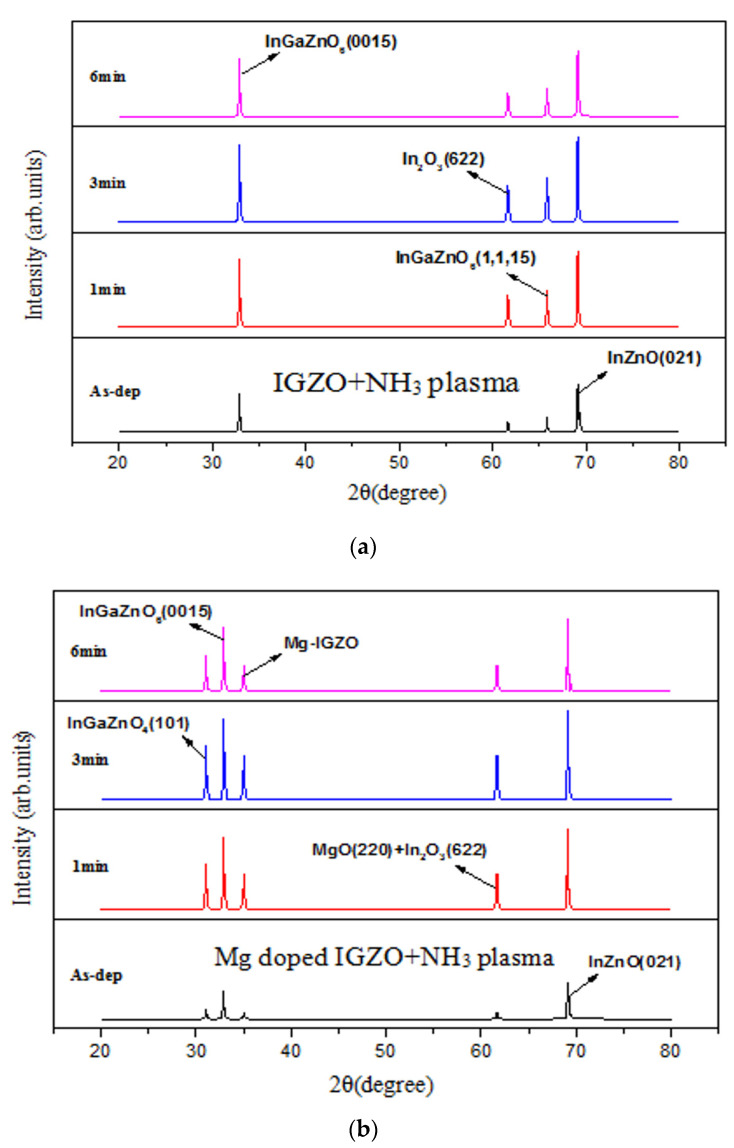

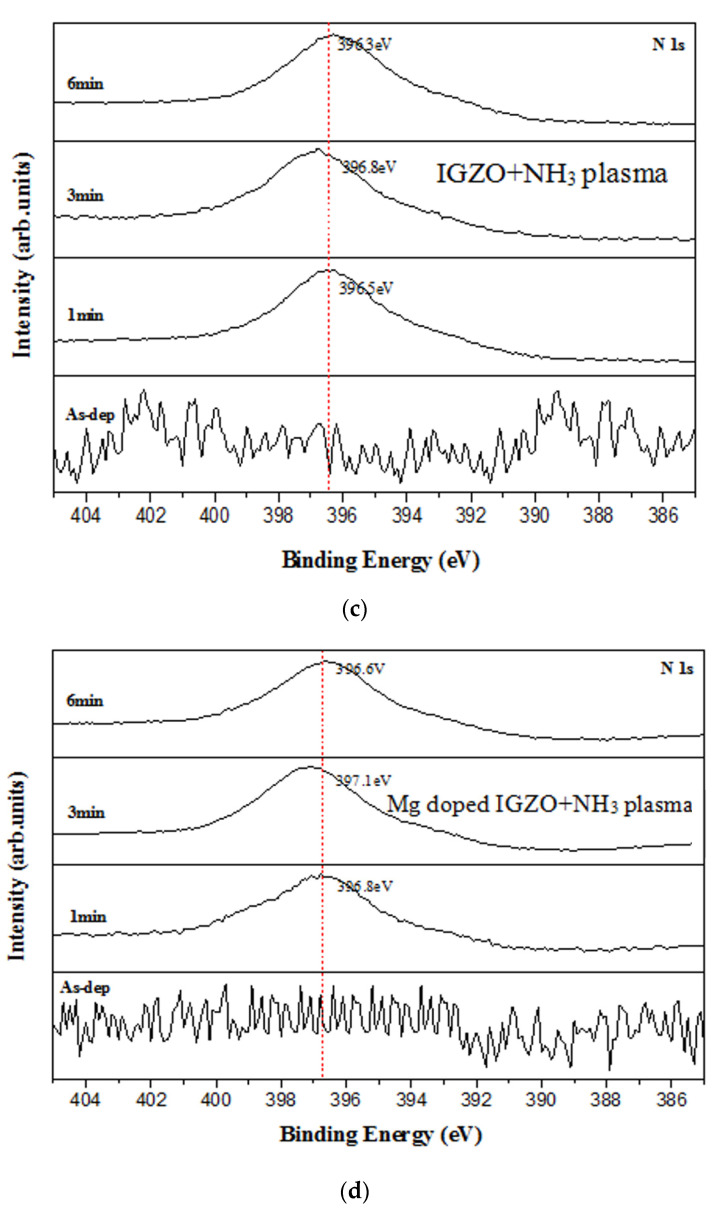

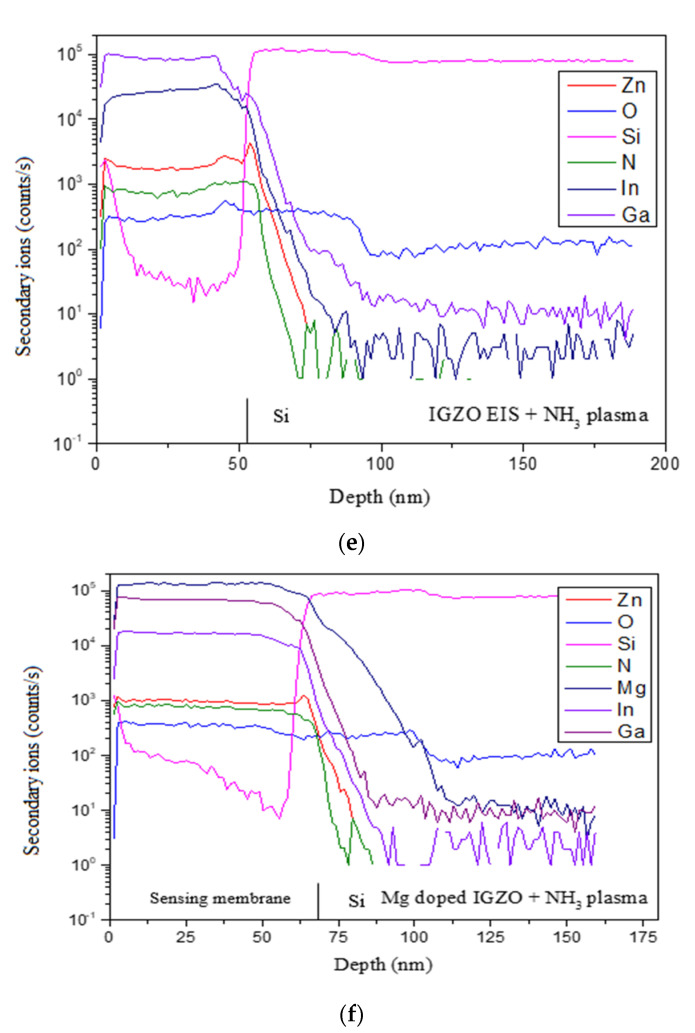
XRD patterns for (**a**) IGZO with NH_3_ plasma treatment and (**b**) Mg doped IGZO with NH_3_ plasma treatment. N 1s XPS spectra for (**c**) IGZO with NH_3_ plasma treatment (only noise for the as-deposited sample: not shown) and (**d**) Mg doped IGZO with NH_3_ plasma treatment. (Various plasma treatment times are included in (**a**–**c**), (only noise for the as-deposited sample: not shown) and (**d**). SIMS data for (**e**) IGZO with NH_3_ plasma treatment for 3 min and (**f**) Mg doped IGZO with NH_3_ plasma treatment for 3 min.

In addition to studying morphologies, XRD, XPS, and SIMS were used to investigate the crystalline phases, chemical bindings, and incorporated atoms versus depth, respectively [4]. Figure 5a,b show XRD patterns for the undoped InGaZnO and Mg-doped InGaZnO membranes with various NH_3_ plasma treatments, showing that Mg-doping and plasma treatment could enhance the crystalline phase. The strongest crystallization occurred in the 3-min plasma treatment of the Mg-doped samples. As the plasma treatment time increased to 6 min, the crystalline phase became weaker. In addition, Mg co-sputtering can form magnesium oxide (MgO) crystals into IGZO as shown in Figure 5a,b and therefore, the sensing behavior can be improved [30,31]. The electronegativity of Mg (χ = 1.31) is low and stablize the binding with oxygen [32]. Moreover, magnesium atoms can replace the vacancies and repair dangling bonds to passivate defects in oxides [33]. Therefore. the membrane films with better material quality can have better pH sensing capability.

XPS spectra for the undoped and Mg-doped samples shown in Figure 5c,d show that an N-chemical binding peak emerged with the NH_3_ plasma treatment. Moreover, the strongest N-binding occurred at 3 min of plasma treatment for both the undoped and Mg-doped samples. This analysis is in line with the XRD patterns. Furthermore, SIMS data revealed that the incorporation N and Mg atoms along the depth of the undoped and Mg-doped samples, as shown in Figure 5e,f. Results indicate that N atoms could be uniformly distributed along the depth of the membrane to passivate defects, as shown in Figure 5e,f. Moreover, high concentrations of Mg could be found in the Mg-doped membrane. SIMS analysis of the showed that In, Ga, and Zn distribution spiked near the membrane/Si interface were mitigated and the defects were suppressed in the doped samples, likely due to Mg doping. This suppression may mitigate defects near the interface, and hence further boost sensing behaviors for the Mg-doped membrane [5].

After morphological and material characterizations, sensing behaviors of the undoped and Mg-doped membranes with NH3 plasma treatment were evaluated [17]. To evaluate the pH sensing behavior of a sensing membrance C-V curves were taken. The capacitance changed with gate bias voltage swept for 3 V interval. As a reference capacitance of 0.4 Cmax is set, the values of reference voltages versus pHvaues could be extracted from the C-V curves. The pH sensitivity and linearity could be calculated from the subfigure of reference voltages versus pH values. The pH senesing evaluated from C-V curves.

**Figure 6:**
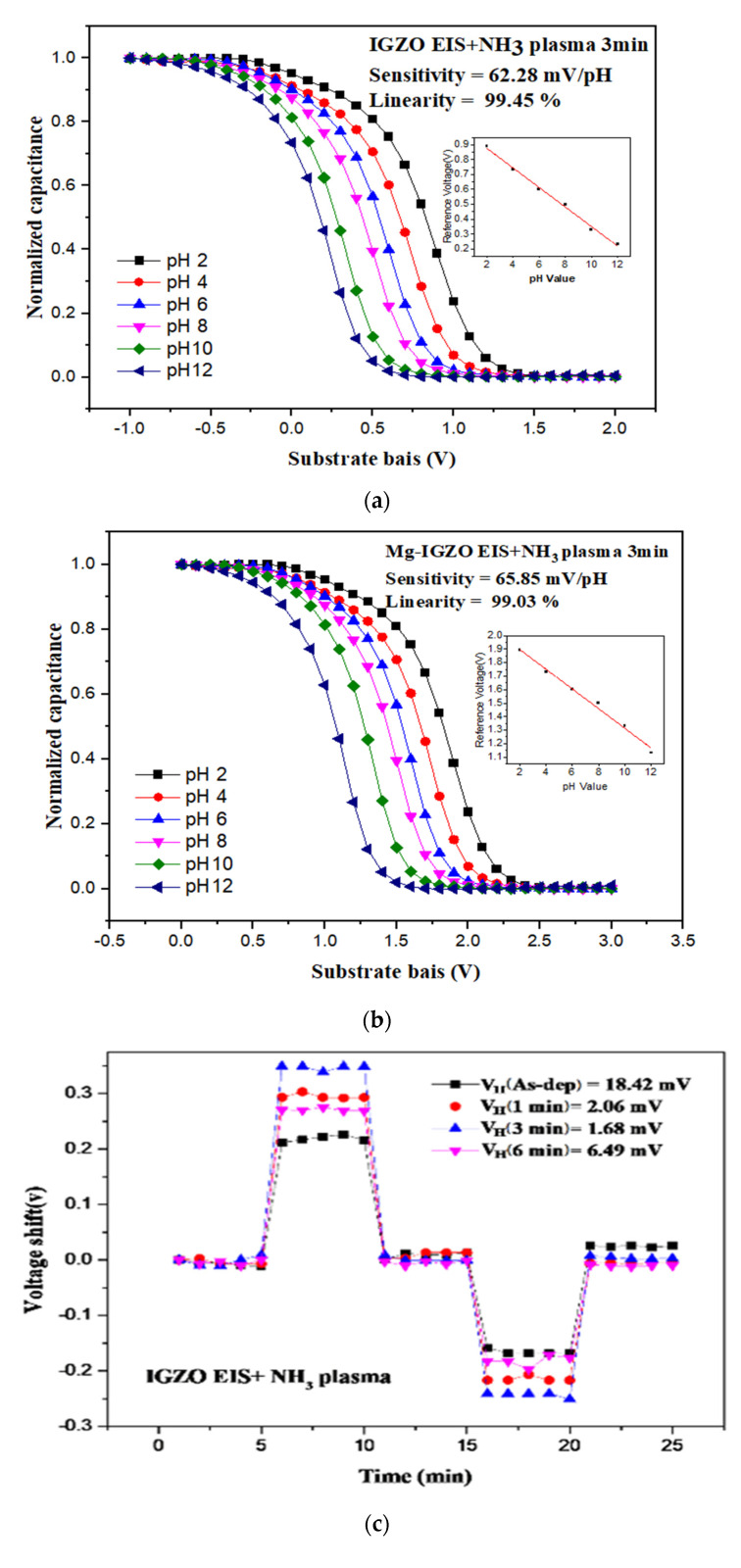

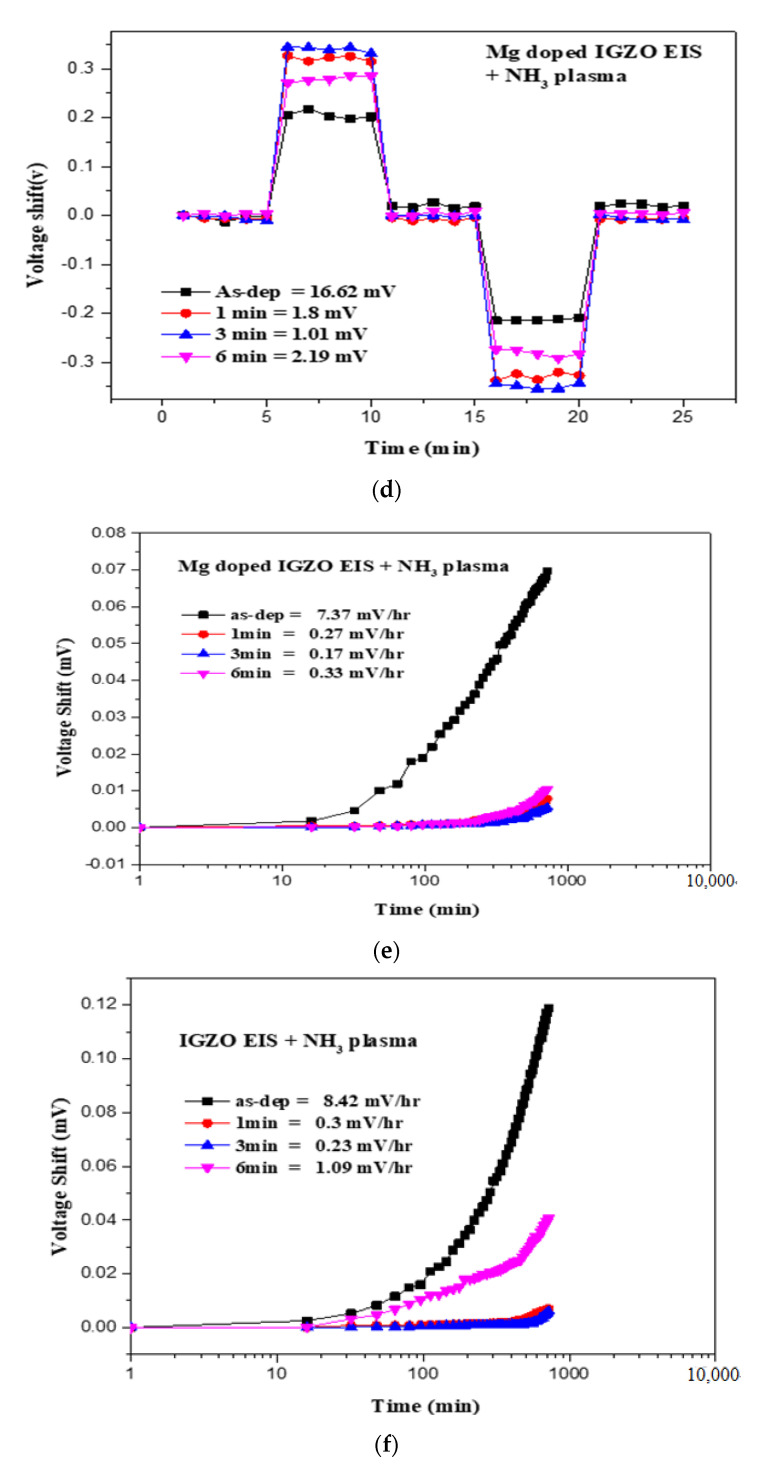
The C-V curves and the extracted pH sensitivity and linearity of the sensing data of (**a**) IGZO with NH_3_ plasma treatment for 3 min and (**b**) Mg doped IGZO with NH_3_ plasma treatment for 3 min. The hysteresis voltage measurements for (**c**) IGZO with NH_3_ plasma treatment for 3 min and (**d**) Mg doped IGZO with NH_3_ plasma treatment for 3 min. The drift voltage measurements for (**e**) IGZO with NH3 plasma treatment for 3 min and (**f**) Mg doped IGZO with NH_3_ plasma treatment for 3 min.

Figure 6a,b show the sensing capability measurements for the undoped and Mg-doped membranes with NH_3_ plasma treatment for 3 min. Results indicate that the pH sensing sensitivity for the undoped InGaZnO membrane achieved 62.28 mV/pH, which was above Nernst limit. However, the sensitivity of the Mg-doped membrane was as high as 65.85 mV/pH, indicating that both Mg doping and NH_3_ plasma treatment could effectively enhance crystallization, reduce defects in the bulk and interface, and reinforce membrane material quality. As shown in Table 1, the sensitivity of NH_3_ plasma treated InGaZnO sample and Mg doped InGaZnO sample have pH sensitivity of 62.28 and 65.85 mV/pH, which are above Nernst limit (around 60 mV/pH), while the InGaZnO and Mg-doped InGaZnO sample with appropriate annealing at 500 °C have the pH sensitivity of 56.51 and 59.3 mV/pH, which are below Nernst limit. Compared with plasma treatment with other gases, NH_3_ might be more effective. Since nitrogen (N) was similar to oxygen (O) in regard to ionic radius and acts as a better compensator, NH_3_ plasma was used in the post-treatment of sputter film. Therefore, we investigated the impact of NH_3_ plasma treatment on the sensing behavior, surface morphology, and crystal structure. Due to the NH_3_ plasma treatment, plasma-induced morphological changes and increment of grain size were observed, favoring the increase of surface roughness and number of surface defect sites, and thus resulting in higher sensitivity and linearity. The surface charge density was mainly related to the ionic activity in the solution.

To assess the stability of the tested membranes, the hysteresis voltages and the drift effect were evaluated. To investigtate the hysteresis effects, the membranes were immersed in solutions with various pH values of 7, 4, 10, and 7 in an alternate time sequence. The submerging time was five minutes in each solution. The hysteresis voltage could be calculated by the voltage deviation between the initial and the terminal voltages taken in the pH loop. The dangling bonds could bind with the ions in the solutions, hysteresis response could be observed.

Hysteresis voltage measurements were then taken for the undoped and Mg-doped membranes, shown in Figure 6c,d, respectively, show that NH_3_ plasma treatment with a time of 3 min can effectively suppress hysteresis voltage, possibly from the removal of the dangling bonds and traps [34]. Compared with the as-deposited InGaZnO film and the as-deposited Mg doped film, the NH_3_ plasma treatment could lower the hysteresis voltage from 18.42 to 1.68 eV for the undoped sample and from 16.62 to 1.01 eV for the Mg-doped sample, respectively.

**Figure 7:**
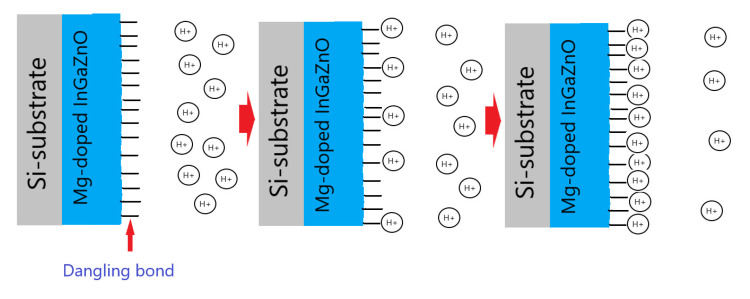
The mechansim for hysteresis and drift voltage shifts caused by dangling bonds.

Finally To examine the drift rate voltage for long-time reliability, the samples were submerged in a pH7 buffer solution for 12 h. Consistent with the material and sensing characterizations, drift voltage rate measurements for the undoped and Mg-doped samples revealed that NH_3_ plasma treatment could effectively remove the defective bindings as shown in Figure 6e,f. Plasma treatment could potentially cause the membrane to bind with ions in the solution. Moreover, Mg doping could further lower both the hysteresis voltage and drift rate, since incorporated Mg atoms might fill in the vacancies and bind with dangling bonds to enhance material quality. The drift voltage shift could also be induced by the ions captured by the dangling bonds, too. To illustrate the voltge shifts in the hysteresis and drift voltage measurements, Figure 7 explain the mechansim for hysteresis and drift voltage shifts caused by dangling bonds. In the beginning, no ions were attached on the dangling bonds. As the measuring time passed by, more and more ions were captured by the dangling bonds and the gate voltage shifts might occur as shown in Figure 7.

**Figure 8:**
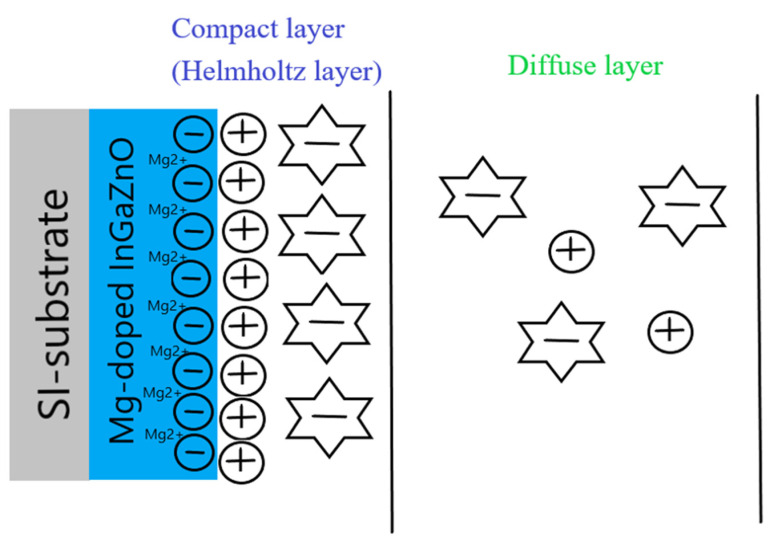
The C_DL_ decrease caused by Mg doping in the site-binding model.

Based on previous reports [23,35,36,37], Mg doping can generate Mg^2+^ near the surface of the membrane and attract some OH^−^ ions in the electrolyte solution. Therefore, the OH^−^ in the diffusion layer of the electrolyte solution might decrease, and therefore the C_DL_ (diffusion capacitance) might decrease as well. The illustration of C_DL_ decrease is shown in Figure 8. Then, the membrane parameter β, the surface potential, and overall sensitivity could also be boosted. However, though NH_3_ plasma treatment could incorporate N atoms into the membrane and potentially decrease defects, it could not cause Mg atoms in the membrane to be uniformly distributed in the same way that Rapid Thermal Anneaing (RTA) annealing did in our previous research. Therefore, linearity could be decreased due to the uneven distribution of Mg atoms with the plasma treatment. In the future, a combination of RTA annealing and NH_3_ plasma treatment could be conducted to further improve the sensitivity and linearity of the membrane. As the plasma treatment time increased to 6 min, the hysteresis voltage and drift rate increased drastically. Since plasma treatment could damage the film by etching away the surface, plasma treatment with a time longer than 6 min could deteriorate the surface material quality consistent with the XRD analysis as shown in Figure 5. Therefore, plasma treatment with an appropriate time could optimize the material properties and device performance.

## 4. Conclusions

In this study, Mg doping and NH_3_ plasma treatment were incorporated into the fabrication process of InGaZnO membranes. Inclusion of Mg and N atoms into the membrane could enhance crystallization, strengthen chemical binding, and reduce defects, as revealed in morphological and material characterizations. Evaluation of the sensing behavior also indicated that Mg doping and NH_3_ plasma treatment with a time of 3 min could boost the sensing behaviors above the Nernst limit, and also enhance low hysteresis voltage and drift rate. Our results indicate that Mg-doped InGaZnO membranes with NH_3_ plasma treatment show promise for future industrial pH sensing EIS-based biosensors.

## Figures and Tables

**Table 1:** Comparison of InGaZnO and Mg doped InGaZnO samples with annealing at 500 °C and NH_3_ plasma treatment for 3 min.

Sample	Sensitivity	Linearity
IGZO w/o annealing	39.08 mV/pH	95.37%
Mg-doped IGZO annealed at 500 °C	56.51 mV/pH	98.79%
Mg-doped IGZO w/o annealing	43.45 mV/pH	99.234%
Mg-doped IGZO annealed at 600 °C	59.3 mV/pH	99.128%
IGZO NH_3_ plasma 3 min	62.28 mV/pH	99.45%
Mg-doped IGZO NH_3_ plasma 3 min	65.85 mV/pH	99.03%

## Data Availability

The data used to support the findings of this study are available from the corresponding author upon request.
